# Modeling the development of coarse-to-fine processing in the central visual pathway

**DOI:** 10.1186/1471-2202-14-S1-P294

**Published:** 2013-07-08

**Authors:** Jasmine A Nirody

**Affiliations:** 1 Biophysics Graduate Group, University of California, Berkeley, Berkeley, CA, 94720, USA

## 

The mode of information processing in neural sensory systems has been the subject of many experimental and computational studies. In the visual system, the existence of a sequential analysis of information, where coarse features are processed before fine details, is intuitive--when looking quickly at a scene, we process general structures before focusing on individual objects. Recent studies have shown that cortical spatial coarse-to-fine processing is largely accounted for by feedforward input from the lateral geniculate nucleus (LGN) in adults [1]. However, the developmental aspect of this process has not yet been considered. We use experimental data from LGN neurons from three age groups: adult cats and kittens at 4 and 8 weeks postnatal [2] to construct a firing-rate based thalamocortical model. We consider how developmental changes in spatiotemporal structure affect feedforward thalamic contribution to cortical spatial frequency (SF) tuning, as well as the efficacy of cortical feedback in facilitating this dynamic. Our model predicts a non-monotone relationship between relative surround strength and shift in tuning peak, suggesting the existence of an optimal center-surround balance which maximizes SF shift. Taken together with previous data, our results suggest that the ratios for mature animals are distributed close to or around their "optimum", while the distribution for kittens is centered at a ratio significantly lower than their peak value. As cortical feedback has been suggested to strengthen the antagonistic effects of the surround response, this implies that kittens early in the developmental process are more strongly affected by corticothalamic feedback than adults. Our results point to these recurrent connections as an important mechanism in facilitating the development of spatial coarse-to-fine processing.

**Figure 1:**
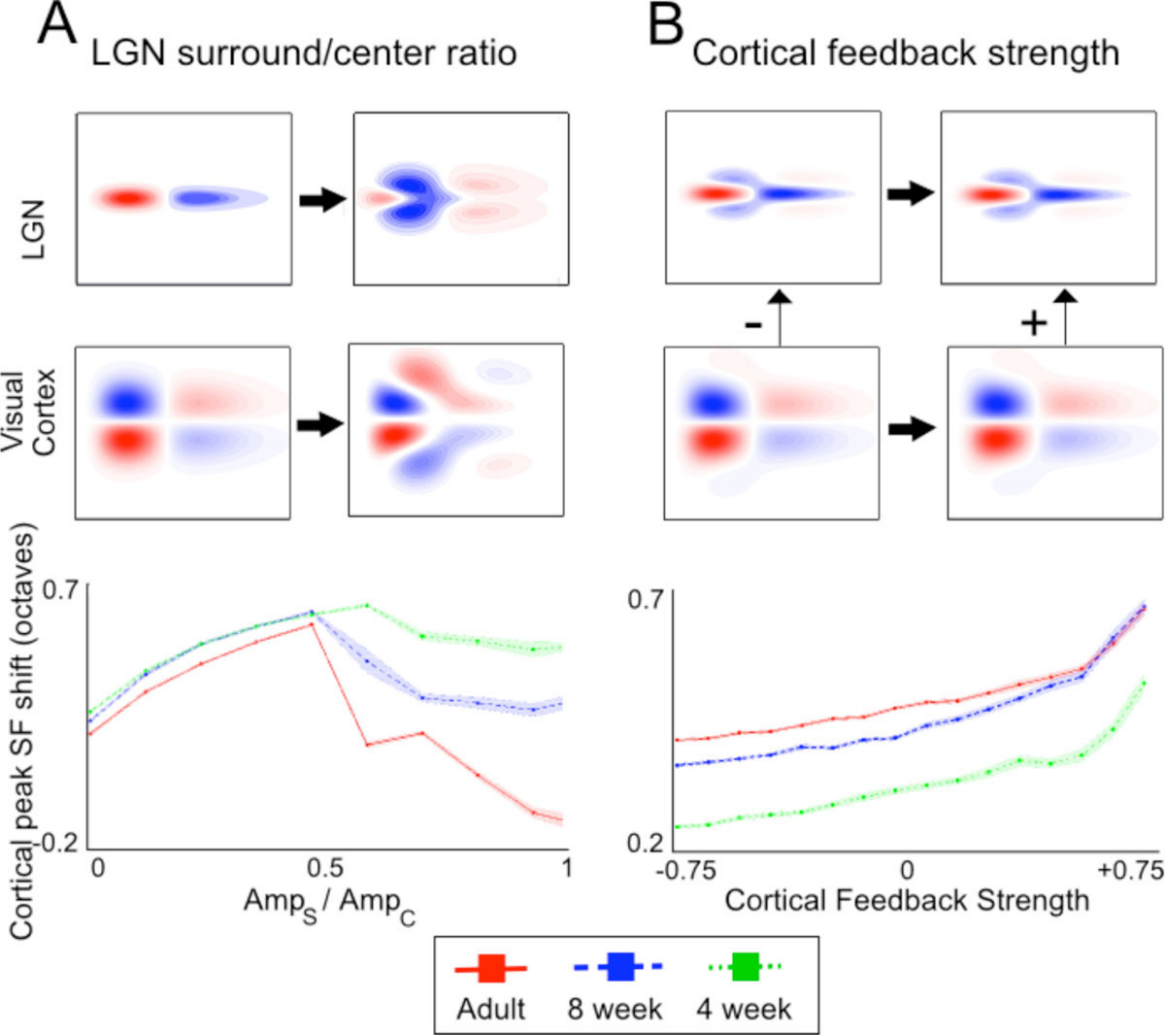
**Effects of selected model parameters on SF tuning in the visual cortex**. Top panels show representative LGN and cortical spatiotemporal receptive fields at low and high ends of the intervals considered. Plots (bottom) show cortical peak SF shift for various values of parameters for each age group. Each point corresponds to the mean taken from 100 simulations, and shaded regions enclose ±1 SEM away from the mean. Center-surround delays are taken from experimental data [2] (8, 12, 16 ms for adults, 8- and 4-week old kittens, respectively). (A) Cortical peak SF shift as a function of surround-center ratio. There was no cortical feedback in this set of simulations. (B) Cortical peak shift as a function of cortical feedback strength *C*. The spatial spread of the feedback *a *was fixed at 0.075. Surround-center ratios were 0.3, 0.2, 0.1 for adults, 8- and 4-week old kittens, respectively [2].
